# EEG Biomarkers in Children and Adolescents With Feeding and Eating Disorders: Current Evidence and Future Directions

**DOI:** 10.3389/fpsyt.2022.882358

**Published:** 2022-04-08

**Authors:** Cristina Berchio, Susanne Cambi, Edoardo Pappaianni, Nadia Micali

**Affiliations:** ^1^Department of Psychiatry, Faculty of Medicine, University of Geneva, Geneva, Switzerland; ^2^Division of Child and Adolescent Psychiatry, Department of Child and Adolescent Health, Geneva University Hospitals, Geneva, Switzerland; ^3^Great Ormond Street Institute of Child Health, University College London, London, United Kingdom

**Keywords:** EEG, ERP, eating disorder, anorexia nervosa, feeding disorders, children, adolescents

## Abstract

**Introduction:**

Electroencephalography (EEG) represents a powerful tool to detect abnormal neural dynamics in child and adolescent psychiatry. Feeding and Eating Disorders (FEDs), such as anorexia nervosa (AN), bulimia nervosa (BN), binge eating disorder (BED), and avoidant restrictive food intake disorder (ARFID) onset in childhood and adolescence. EEG has rarely been used to examine cortical brain activity in children and adolescents with FEDs. This review aims to summarize EEG findings in FEDs amongst children and adolescents, and to highlight areas deserving further research.

**Methods:**

We searched the literature for EEG studies on children and adolescents with FEDs using Google Scholar, PsycINFO, Medline, and PubMed.

**Results:**

Twelve studies were identified, the majority focusing on AN (*N* = 10). The identified studies suggest reduced action monitoring control (preparatory waves, N200, P300), specific perceptual-cognitive styles to body/face perception (late positive potentials/early posterior negativity), as well as fundamental changes in posterior theta oscillations in AN. Behavioral traits of BN/BED (i.e., loss of control eating, emotional eating), and AN seem to be associated with an increased attentional reactivity (P300) to visual food stimuli.

**Conclusion:**

Electroencephalography research in children and adolescents with FEDs is limited and mostly focused on AN. While EEG abnormalities seem consistent with a reduced top-down control and attentional allocation deficits in AN, altered attention specific to food cues emerges across FEDs. Overcoming conventional EEG analyses, and investigating spatial properties (i.e., electrical neuroimaging), will enhance our understanding of FEDs neurobiology.

## Introduction

Electroencephalography (EEG) captures cortical electrical activity in the order of milliseconds, and electrical neuroimaging methods allow to describe underlying large-scale brain networks with good spatial resolution (1). EEG is a non-invasive technique particularly suitable for the investigation of brain functions in child and adolescent psychiatry (2). The main advantage of applying EEG in this domain is the possibility of performing cognitive assessments in semi-ecological contexts, by implementing procedures that are tolerated by the majority of clinical populations.

Feeding and eating disorders (FEDs) can cause serious health problems to children and adolescents (3). Anorexia Nervosa (AN), Bulimia Nervosa (BN), Binge Eating disorder (BED), and Avoidant Restrictive Food Intake Disorder (ARFID) onset in childhood and adolescence (4). Intense fear of gaining weight, distorted body perception, and abnormally low body weight are characteristic of AN, whilst eating large amounts of food and experiencing loss of control (binge eating) is a key feature of BED; BN is defined by binge eating and compensatory behaviors (i.e., vomiting, laxative use) (5). ARFID is an extreme form of picky or selective eating associated with sensory sensitivity, lack of interest in food, and fear of aversive consequences, with physical and psychosocial impact (6). The detection of brain signatures of FEDs is key in developing treatments for these disorders in youths.

Results from EEG research consistently demonstrate neural oscillatory abnormalities in individuals diagnosed with FEDs, specifically in beta bands in BED and BN (7), and in theta bands in AN (8, 9). Event-related potentials (ERP) are stimulus-locked potentials, captured through averaging procedures, that reflect cortical responses to external cognitive, sensory, or motor events (10). ERP studies show consistent attentional bias toward food pictures in adults with abnormal eating behaviors (11). Nevertheless, EEG has rarely been used to examine cortical brain activity in children and adolescents with FEDs.

Identifying brain signatures of FEDs can help prevent the development of these disorders, and refine diagnosis and treatments strategies. This review aims to provide an overview of EEG biomarkers of FEDs in children and adolescents by briefly presenting the available literature and, by highlighting findings, gaps, and future directions that may aid research. We will focus on ERP and EEG oscillations and on the extent to which both are affected by eating disorders.

## Materials and Methods

### Strategy and Eligibility Criteria

**FIGURE 1:**
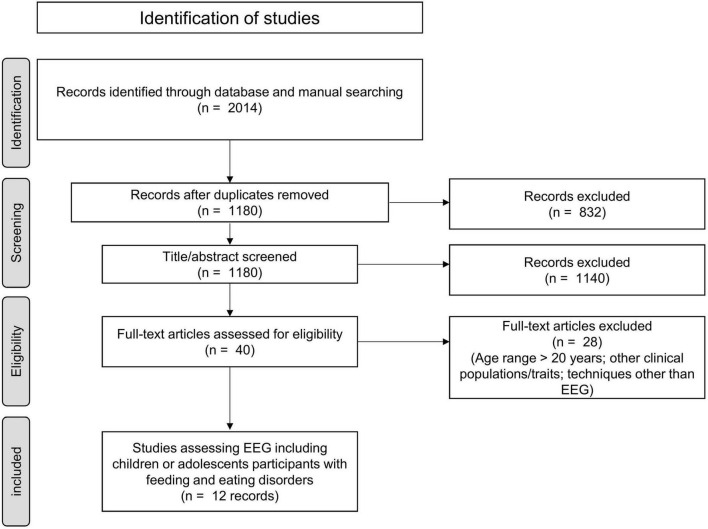
PRISMA flow chart for study selection.

The review was conducted using PubMed, SCOPUS, Google Scholar, and PsycINFO. Studies were included if they: (1) studied children or adolescents participants with FEDs or related symptoms and; (2) used EEG with a task protocol or spontaneous activity. Articles were required to be published in a peer-reviewed journal and to be written in English. Search terms included: anorexia nervosa OR bulimia nervosa OR binge eating disorder OR avoidant restrictive food intake disorder AND EEG OR ERP AND children OR adolescents. Final searches were conducted in January 2022. The selection of the studies was conducted according to the PRISMA protocol (12). Figure 1 shows the PRISMA flowchart with results in each categorization step.

Articles searches were carried out by SC and CB. Twelve studies were included in this review (Table 1). From each study, the following information was extracted: FEDs diagnosis or symptoms, age, sample size, gender, paradigm used, stimuli, and EEG outcome measures.

**TABLE 1:** Summary of findings.

Study	Clinical population/traits	Participants	Paradigm	EEG measure	Main EEG findings	Study quality assessment
**EVOKED POTENTIALS**						
**Executive control**						
Bradley et al. (21)	AN (females)	AN, *N* = 20 (mean age 15.7), 8 re-tested after weight gain; HC, *N* = 20 (mean age 15.7).	Verbal and non-verbal memory task	P300	P300 abnormalities in AN compared to controls, that persist after weight gain.	75%
Yue et al. (23)	AN (females)	AN, *N* = 27 (mean age 19 (adolescents and adults); HC, *N* = 30 (mean age 19.23).	Stop-signal task	P300, N200	Lower P300 amplitudes and longer N200 latencies to response inhibition in AN.	75%
Torigoe et al. (24)	AN (7 females, 1 male)	AN, *N* = 8 (mean age 12.9); HC, *N* = 23 (mean age 12.9).	Warning stimulus + go signal	Slow wave (CNV)	Diminished CNV amplitude in AN.	58%
**Food related processing**						
Novosel et al. (25)	AN (females)	AN, *N* = 11 (mean age 15.78); HC, *N* = 11 (mean age 15.7).	Passive viewing (food and emotions) + rating	P300, LPP	Augmented P300 and LPP to food stimuli in AN.	79%
Biehl et al. (26)	Loss of control eating (9 females, 6 males)	Loss of control eating, *N* = 15 (mean age 12.9); HC, *N* = 19 (mean age 12.3).	Food Go-NoGo task	P300	In loss of control eating, enhanced P300 amplitudes to high-calorie food stimuli (vs. neutral) in NoGo trials.	54%
Wu et al. (27)	Emotional eating (38 females, 48 males)	*N* = 86 (mean age 13.86).	Food and non-food viewing task (mental imagery)	P300, LPP	Augmented P300 and LPP amplitudes for food compared to non-food cues in emotional eating.	58%
**Body and face perception**						
Horndasch et al. (28)	AN (females)	AN, *N* = 13 (mean age 15.7); HC, *N* = 18 (mean age 16.6).	Affective rating of body stimuli	LPP	Augmented LPP amplitudes to pictures of underweight body shapes in AN.	63%
Horndasch et al. (29)	AN (females)	AN, *N* = 32 (19 adolescents, mean age 15.2; 13 adults); HC, *N* = 37 (16 adolescents, mean age 16.3; 21 adults).	Attractiveness and weight rating of body stimuli	LPP	Adolescents with AN and controls show similar late positive potential (LPP) to picture of underweight women.	67%
Sfärlea et al. (30)	AN (females)	AN, *N* = 20 (mean age 15.2); HC, *N* = 24 (mean age 15.7).	Emotional face processing	EPN	Less pronounced early posterior negativity (EPN) in response to all facial expressions in AN.	67%
**NEURAL OSCILLATIONS**						
Hatch et al. (36)	AN (females)	AN, *N* = 37 (mean age 15.16), 28 re-tested after weight gain; HC, *N* = 45 (mean age 14.98).	Resting state	Delta, theta, alpha, beta	Reduced alpha and increased beta, theta in underweight AN. After weight gain, elevated theta power.	92%
Lackner et al. (15)	AN (females)	AN, *N* = 22 (Experimental group = 12, Control group = 10) age range 12–18.	Resting state/Neurofeedback	Theta, alpha, beta	Increase in theta power from pre to post neuro-feedback.	88%
Grunwald et al. (37)	AN (females)	AN, *N* = 10 (mean age: 15.9), 10 re-tested after weight gain; HC, *N* = 10 (mean age: 16.14).	Resting state/Haptic exploration	Theta	Reduced theta power in AN during haptic exploration.	54%

*AN, anorexia nervosa; HC, healthy controls; CNV, contingent negative variation; LPP, late positive potential; EPN, early posterior negativity.*

### Study Quality Assessment

The risk of bias in individual studies was assessed using the 14-item Kmet checklist (13). However, since 3 criteria did not apply to the main aim of this review (i.e., random allocation to the treatment group, blinding of investigators, blinding of subjects), only 11 items were used. Each item received a score of 2 if the question was sufficiently described, 1 if it was only partially described, 0 if it was not addressed. Based on these scores, percentage values were calculated, and studies were classified as strong (scores > 80%), good (70–80%), adequate (50–69%), or limited (scores < 50%) [see (14)]. Two authors carried out the rating independently.

## Results

### Study Characteristics

After full-text examination, 12 studies were included (see Table 1). Ten include individuals with AN, one adolescents with emotional eating, and one adolescents with loss of control eating.

The 12 selected studies showed a mean sample size of 24 (SD ± 21.19) participants. The largest study included 86 participants, the smallest 8. The mean age of participants was 15.21 (SD ± 1.68) years, and one study only reported the age range of the sample investigated (15). In relation to gender, 81.25% of participants included were females (234 females and 54 males).

One study was considered of strong methodological quality (score > 90%), four of good quality (75–87.5%), and seven of adequate quality (54–67%), as assessed by the Kmet checklist. Limitations primarily involved sample size, and lack of controlling for confounding variables.

Nine studies capitalized on ERP methodologies, while three studies employed neural oscillations analysis. Due to differences in methodology and research questions, the results from these studies are presented separately.

### Synthesis of Results

Studies were sorted and summarized according to the EEG marker of interest: ERP and neural oscillations. Within the ERP group, studies were classified into the following subcategories: executive control, food-related processing, body and face perception.

#### Event-Related Potentials

Event-related potentials components are classically defined by positive (P) or negative (N) polarity, latency, and stimulus-induced activity (10). Early components are believed to reflect neuronal sensory processing (e.g., P100), categorization (e.g., N200), brain encoding of information (e.g., N170), while later components reflect higher order cognitive processing, such as attention (e.g., P300, late positive potential “LPP”), emotional decoding (e.g., early posterior negativity “EPN”), and semantic memory [see (16–18)]. Slow waves represent an additional family of evoked responses, induced by cognitive (e.g., contingent negative variation “CNV”) and/or motor preparation (19, 20).

##### Executive Control

Three studies measured executive control in individuals with AN. In the study by Bradley et al. (21), adolescents with AN completed two memory tasks (one verbal and one non-verbal with abstract figures) during the EEG recording. No significant differences were found in behavioral measures of the task. However, within the AN group, ERP data indicated significantly longer N400 latencies for the non-verbal task than for the verbal task, moreover a positive correlation between body mass index (BMI) and N400 amplitude was found for the verbal memory task. Between groups, individuals with AN showed longer P300 latencies in the verbal task. For the non-verbal task, P300 amplitudes were positively correlated with BMI, and negatively correlated with dieting/thinness scores [on the Eating Attitudes test-EAT (22)] and with depressive symptoms. P300 amplitude abnormalities were also observed in 8 out of 20 weight restored individuals with AN in a follow-up assessment.

Yue et al. (23) examined response inhibition in AN patients and healthy controls using a stop-signal paradigm. ERP were obtained for three different stop-signal delays. AN patients showed lower accuracy, lower P300 amplitudes, and longer N200 latencies compared to controls. Only in the AN group, N200 latencies were sensitive to the stop-signal time delay; furthermore, the amplitude of the N200 was positively correlated with ED symptoms (22), whilst the N200 latency and P300 amplitude were positively correlated with BMI.

Torigoe et al. (24) measured CNV during a task involving an auditory warning stimulus and an imperative stimulus (flashes), to which children had to respond by pressing a button. No significant behavioral differences were observed between the AN group and controls. Early and late CNV were significantly smaller in AN children than in controls.

##### Food Related Processing

Three studies examined food related-processing in youth with FEDs (see Table 1).

In a study by Novosel et al. (25), adolescents with AN and controls were exposed to pictures belonging to five categories (pleasant/unpleasant items, high/low-calorie food, neutral objects) and were asked to rate them on arousal and valence. AN patients rated food pictures as less pleasant and more arousing, and showed higher P300 and LPP amplitudes to low-calorie food pictures compared to controls.

Biehl et al. (26) investigated visual processing and inhibition in adolescents with loss of control eating (at least one episode of loss of control eating in the last 4 weeks) and a matched control group. Participants completed a color-based Go/NoGo task. The stimuli were numbers of one of two colors (Go vs. NoGo), presented side by side with distractors (high-calorie food or neutral stimuli). No behavioral differences between groups were found. In NoGo trials, the loss of control eating group, but not the control group, showed significantly higher P300 amplitudes when high-calorie food distractors were presented, compared to neutral stimuli.

In a study on emotional eating traits (27), female and male adolescents performed an EEG task with food (high-fat foods, savory snacks, sweets) and non-food stimuli. Participants were instructed to try and imagine the taste of each food. Food stimuli, compared to non-foods, elicited higher amplitudes on both the P300 and the LPP. In fronto-central regions, the LPP difference wave (food stimuli vs. non-food stimuli) was positively correlated with emotional eating. Source localization of the LPP to food stimuli indicated a maximum in the orbito-frontal cortex.

##### Body and Face Perception

Three studies have examined body and face perception in individuals with AN. In a study by Horndasch et al. (28), participants were exposed to pictures of unclothed bodies in three different weight categories (underweight, normal-weight, and overweight). Participants were asked to rate their emotions (fear, disgust, and happiness). No differences in ratings were identified between groups. Adolescents with AN showed the highest LPP amplitudes when exposed to underweight bodies, followed by normal-weight and overweight ones. A reverse pattern of LPP amplitudes was observed in controls.

In a subsequent study by Horndasch et al. (29), adults and adolescents with AN viewed pictures of women’s bodies in underwear belonging to extreme BMI categories. Participants rated the pictures on attractiveness and weight. Behavioral ratings showed higher attractiveness for extremely under-weight bodies in AN compared to controls. However, no ERP differences were found between groups.

Sfärlea et al. (30) used a passive viewing task, and three discriminative active tasks on face-word, gender, and emotion discrimination to investigate the neural correlates of emotional face processing in youth with AN and controls. No behavioral impairments were identified. However, the AN group showed a less pronounced EPN negativity independently of facial expressions and across all tasks compared to controls.

#### Neural Oscillations

Neural oscillations can be observed in EEG signals in the frequency domain and are classified, according to their frequency range, into: delta (0–4 Hz), theta (4–7 Hz), alpha (approximately 10 Hz), beta (14–30 Hz), and gamma waves (above 40 Hz) (31). Different bands are associated with top down cognitive control [e.g., posterior alpha and anterior theta (32, 33)], sensory-motor modulations [e.g., central alpha (34)], and affective states [e.g., frontal theta (35)].

Three studies have looked at neural oscillations in adolescents with AN, two of these aimed to examine resting state activity (36), and sensory integration (37) using a longitudinal design. Hatch et al. (36) collected EEG data in AN patients and controls in “eyes-open” and “eyes-closed” conditions. In the “eyes-closed” condition, AN patients showed reduced alpha power in frontal regions and elevated theta power in parietal-occipital sites when compared to controls. In the “eyes-open” condition, individuals with AN showed an overall reduced alpha power, and an enhanced beta power in frontal regions compared to controls. Following weight restoration delta, alpha and beta abnormalities were restored, while the increased theta power during “eyes-closed” persisted in youth with AN. Theta power has also been assessed during resting-state and during a haptic exploratory task in individuals with AN and controls. In both conditions participants kept their eyes closed. Both groups showed an overall decreased theta during haptic exploration vs. rest. Group comparisons on haptic exploration indicated that theta power was lower in right posterior regions in AN patients compared to controls, both before and after weight restoration (37).

Resting state activity in AN patients has also been investigated indirectly, by means of neurofeedback (15). Over a period of 5 weeks, AN patients underwent a neurofeedback training aimed at enhancing alpha power. Three minutes of eyes closed and eyes open resting-state EEG were collected pre- and post-training. The authors reported a significant increase post-training in theta power during eye-closed, while no significant effects were found for alpha power. The high theta power at post-training was associated with decreased emotional distress.

### Summary of Findings

Event-related potentials findings on executive control point to a reduced function of neuronal circuits involved in action monitoring (anticipatory control, memory, inhibition) in youth with AN. Furthermore, enhanced attentional mechanisms to food stimuli appear to be a shared feature across a spectrum of eating/feeding disturbances. ERP data on body and face processing suggest enhanced attentional mechanisms to underweight body schemas, driven by emotional processes, as well as a reduced sensitivity to facial emotional expressions.

**FIGURE 2:**
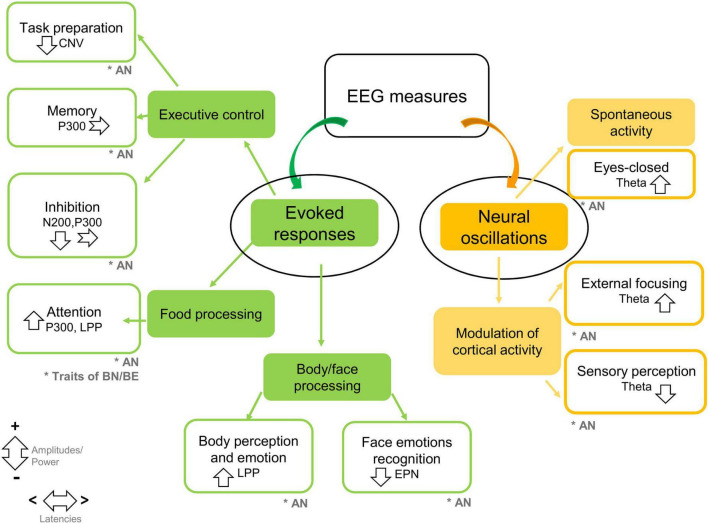
Summary of EEG findings in feeding disturbances in children and adolescents. Studies are grouped by methodologies of EEG analysis (evoked responses, neural oscillations), and experimental protocols. The main EEG markers and findings are summarized. Down-ward arrows indicate decreased amplitudes/power; upward arrows indicate increased amplitudes/power. Horizontal arrows indicate shorter/longer latencies (CNV, contingent negative variation; EPN, early posterior negativity; LPP, late positive potential; AN, anorexia nervosa; BN, bulimia nervosa; BN, binge eating). The symbol * means target population.

Studies on neural oscillations point to abnormalities in posterior theta power, both during spontaneous brain activity and during sensory exploration, that seem to persist after weight restoration, and are potentially restored by EEG neuro-feedback interventional strategies. All EEG findings are summarized in Figure 2.

## Discussion

The main objective of this study was to provide an overview of EEG findings in the field of child and adolescent FEDs. AN was most commonly studied. The overall evidence in AN seems to indicate reduced executive control, specific perceptual-cognitive styles to body/face perception, as well as fundamental changes in posterior theta oscillations. Studies mainly examined food processing across disorders/eating attitudes. The P300 appears a candidate marker of attentional deficits to food across FEDs.

Consistent with the literature on adult AN (38, 39), ERP evidence in youth with AN indicates a reduced function in neuronal circuits involved in action monitoring. These findings provide precise temporal information on executive dysfunctions at multiple stages of action control: preparation, assimilation of information, and inhibition. While behavioral deficits were only identified for task inhibition, effective performance in working memory and anticipatory control seems to rely on different neural mechanisms in AN vs. healthy controls. Therefore, we could assume that action inhibition, which involves multiple stages of action control, is heavily impacted in AN and results in more pronounced behavioral impairments.

Studies on body and face perception indicate that adolescents with AN show augmented attentional neural responses to under-weight bodies during emotional ratings (28), and reduced neural responses during facial expression discrimination, independently of the type of emotion (30). Interference of both global and local irrelevant information is a central characteristic of weak central coherence in adolescents with AN (40). This model could also be applied to body/face perception. ERP findings in this review suggest that youth with AN process bodies by focusing on local information (i.e., details); but they process faces by focusing on global information (paying less attention to details/emotional expressions). Future studies should investigate the link between ERP and impairments in social functioning in AN.

Feeding and Eating Disorders behaviors seem to be associated with an increased neural reactivity to visual food stimuli (25–27). Enhanced attentional processing of food stimuli may represent a transdiagnostic signature of vulnerability to FEDs, and this may be due to a reduced top-down control of prefrontal regions, and/or to abnormally active reward regions (41, 42). Only one study has investigated brain sources, in a sample of adolescents with emotional eating, highlighting maximal activity in a region involved in reward processing (i.e., the orbito-frontal cortex) (26).

Brain waves have only been studied in adolescents with AN. Increased theta activity after weight restoration was interpreted as a stable feature of AN (36). External manipulations of oscillatory activity, such as a neuro-feedback protocol (15) and haptic exploration (37), showed a high sensitivity to theta frequency in AN (in positive and negative directions). The high theta power at post-training was associated with decreased emotional distress, and reduced theta in haptic exploration was linked to deficits in multi-sensory integration independent of nutritional stages. Findings on theta abnormalities appear consistent with evidence in adults with AN (8, 9), and may indicate that adolescents with AN show a different maturational profile on theta waves, reflecting a reduced regulation of top-down control processes.

Body mass index appears to be positively associated with memory-related neural responses and negatively associated with neural substrates of inhibitory control in AN (21, 23). Furthermore, depressive symptomatology is negatively associated with ERP markers of attention and memory in AN (21). One could speculate that either the severity of AN may be associated with more pronounced impairments related to action control, and with executive dysfunctions, potentially an effect of weight loss at a time of increased brain development.

The main limitations of the current literature are its breadth (the majority of studies focus on AN, and on females), sample size (4 studies have sample sizes smaller than 15 patients) and number of available studies (we only identified 12 studies). This limits the generalizability of findings to all FEDs, as well as their reproducibility. Furthermore, analysis on *a priori* time windows and on specific electrodes/frequency bands may have reduced the ability of capturing additional impairments. Similarly, only one study documented ERP brain sources (27).

## Future Directions

Electroencephalography represents a powerful tool to study brain functions in FEDs. Future studies should focus on other types of FEDs in the young population, using larger sample sizes, gender balance, and a range of EEG methodologies. To date, most studies adopt traditional approaches to EEG analysis, which are susceptible to potential biases (43). Globally, these methodological limitations show that EEG spatial properties have not been fully investigated. Future studies should make use of spatio-temporal methodologies of analysis, to provide further insights into temporal dynamics of large-scale networks.

In conclusion, reduced executive control, abnormalities in social-affective information processing, and altered maturational processes of theta waves may represent traits/biomarkers of adolescent AN. Increased P300 attentional responses to food may represent a biological marker of vulnerability to FEDs. EEG represents an objective method to measure changes in neural activity during brain development and could be a valuable tool for the assessment of clinical outcomes within this population.

## Author Contributions

CB, SC, EP, and NM contributed to conceive and design this review. CB and SC conducted the selection of studies, classification of articles, and writing of the manuscript. CB took care of the technical aspects of the manuscript related to EEG. NM carefully supervised the aspects on eating disorders. All authors contributed to the interpretation of data and to the final version of this article.

## Conflict of Interest

The authors declare that the research was conducted in the absence of any commercial or financial relationships that could be construed as a potential conflict of interest.

## Publisher’s Note

All claims expressed in this article are solely those of the authors and do not necessarily represent those of their affiliated organizations, or those of the publisher, the editors and the reviewers. Any product that may be evaluated in this article, or claim that may be made by its manufacturer, is not guaranteed or endorsed by the publisher.
